# Influenza A virus NS1 suppresses nuclear speckles promoted gene expression by inhibition of transcription

**DOI:** 10.1038/s44298-025-00124-x

**Published:** 2025-05-30

**Authors:** Wolfgang Nacken, Juliane Mayr, André Schreiber, Stephan Ludwig

**Affiliations:** Institute of Virology (IVM), University of Muenster and University Hospital Muenster, Münster, D-48149 Germany

**Keywords:** Microbiology, Pathogenesis

## Abstract

IAV-NS1 proteins that interact with Cleavage and polyadenylation specific factor 30 are known to inhibit host gene expression. Here we report that in both transfection and infection experiments, a strong attenuation of reporter gene expression is observed when NS1 proteins are fused to protein domains guiding NS1 exclusively to nuclear speckles (NSP). NS1 proteins that are fused to domains that guide them to nuclear or non-nuclear compartments other than NSP show little or no ability to attenuate reporter gene expression. An NSP-localized NS1-effector domain is sufficient to inhibit gene expression. The protein SON is an essential component of NSP. SiRNA-mediated suppression of SON reduced the ability of NS1 to suppress the expression of a reporter gene relative to cells with fully functional NSP. Lastly, we demonstrate that the NS1-mediated suppression relies on transcriptional inhibition. Our data suggest that IAV-NS1 suppresses NSP-promoted gene expression by inhibition of transcription.

## Introduction

NS1 protein is a multifunctional key protein of the influenza virus, acting mainly against host innate immunity. Upon infection, NS1 is strongly expressed and is found in the nucleus and the cytoplasm. It is a major player in the mediation of host gene shutoff in virus infection, which is a key process contributing to the viral takeover of the cellular metabolism. In case of an influenza A virus (IAV) infection, a global transcriptional inhibition and defects at the 3′ ends of active host genes have been observed. This leads to RNA polymerase II (RNAPII) run-through into extragenic regions and to aberrant RNAs 3′ extensions, host-gene fusions, and down of gene transcription (DOG) that ultimately cause global transcriptional downregulation of physiological transcripts, an effect influencing antiviral response and virulence^1–4^. This phenomenon has been confirmed with multiple strains of IAV and is dependent on the effector domain of influenza NS1 protein^2–4^. The early finding that NS1 of most IAV subtypes blocks the binding of the whole CPSF (Cleavage and Polyadenylation Specific Factor) complex to pre-mRNA and thereby inhibits the cleavage and polyadenylation of mRNA^5,6^ as well as the nuclear-cytoplasmic transport and processing of host mRNA^7^ may play a major role in this scenario.

In search of a molecular mechanism, we recently observed that nuclear NS1 is not associated with genomic DNA^4^. This observation suggests that a direct interaction of NS1 with genomic DNA does not induce the deregulation and/or inhibition of transcription. Consequently, if NS1 is not associated with nuclear DNA, the question arises as to how NS1 then contributes to the deregulation of host transcription.

The mammalian cell nucleus is known to be a highly compartmentalized and yet extremely dynamic organelle. Viral infections are known to alter the cell’s entire nuclear architecture to hijack host machinery^8^. Inside the nucleus, there are nuclear bodies, which are membrane-less structures found in the cell nuclei of all eukaryotic cells. One of the most prominent nuclear bodies is the so-called nuclear speckles (also known as nuclear or interchromatin domains, interchromatin granule clusters, or nuclear dots)^8,9^. Several lines of evidence point to nuclear speckles (NSPs) acting as storage, assembly, and/or modification compartments that can supply splicing factors to active transcription sites^10^. Others view speckles as direct transcription/pre-mRNA splicing centers or suggest that they may have a more direct role relating to the splicing and transport of pre-mRNA^11–15^. However, the presence in NSP of many other factors involved in mRNA production by RNA polymerase II also supports their intimate relationship with gene expression^16^. Recent reports stress the roles nuclear speckles may play in enhancing gene expression and describe how gene positioning to specific nuclear landmarks can regulate gene expression and RNA processing^17–19^.

For some of the speckle components, a speckle-targeting signal peptide sequence has been identified. The arginine/serine-rich domain (RS domain) of some SR pre-mRNA splicing factors has been shown to be necessary and sufficient for the targeting of these factors to nuclear speckles^20–22^. Phosphorylation of the RS domain seems to regulate the recruitment of SR proteins from and association with nuclear speckles^23,24^.

We previously observed that an IAV-NS1 (NS1 allele A) from strain SC35M that lacked both native nuclear localization signals (NLS) but was fused with an SV40 NLS inhibited host cell expression similarly to wild-type NS1 and accumulated in granular structures of the nucleus^4^. This prompted us to speculate that nuclear NS1 may associate with some nuclear bodies and may interfere with their functions. It is reported that nuclear speckles (NSP) contain little or no DNA^8^, which would fit with our finding that NS1 does not associate with genomic DNA. Thus, we asked whether the presence of NS1 in NS is essential for the inhibition of host gene expression.

By relating the subcellular localization of recombinant NS1 proteins with their ability to disturb host transcription, our data suggest that the presence of NS1 in the NSP of the nucleus contributes to host gene shutoff by inhibiting NSP-promoted host transcription.

## Results

### NS1 deficient for its native NLS, but fused with an SV40 NLS is localised at granular structures in the nucleus in an IAV infection scenario

**Fig. 1:**
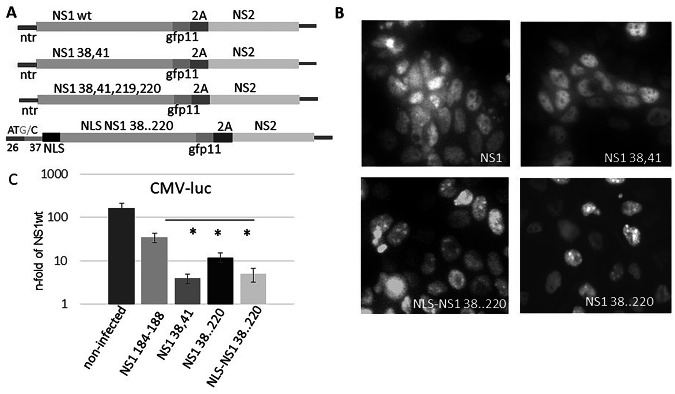
NS1 devoid of its native NLS but fused with a SV40-NLS accumulates in granular structures in the nucleus and inhibits host gene transcription. **A** schematic representation of the NS Segment encoding plasmid used to generate recombinant influenza viruses expressing NS1-GFP11 as indicated. NLS: nuclear localisation site of SV40 Tag; ntr: non-translated region; ATG/C: the first ATG was mutated to ATC to elongate the ntr, which was necessary to enhance virus titre; 26 37: length in base pairs of the sequence homologous to wild type NS segment used to elongate the ntr; GFP11: the 11th domain of GFP superfolder protein; 2 A:2 A peptide from porcine teschovirus-1. **B** Fluorescence microscopy: MDCK cells transduced with pQCXIP-GFP1-10 cells were infected with NS1-GFP11 encoding IAV. Self-assembled GFP fluorescent proteins indicate the localisation of NS1 expressed by the recombinant IAVs. NS1wt: wild type NS1-GFP11; NS1 38,41: NS1-GFP11 (R38A, K41A); NS1 38..220: NS1 38, 41, 219, 220-GFP11 (R38A, K41A, K219A, R220A), NLS-NS1 38..220: NLS-NS1 38, 41, 219, 220-GFP11 (R38A, K41A, K219A, R220A) NS1 184-188: (GLEWN184-188 RFKRY), control- known to be unable to bind CPSF30 and unable to inhibit reporter gene expression. **C** reporter gene assay: infected cells were transfected with CMV-luciferase construct. The luciferase activity of cell extracts (6 h p.i.) from NS1 wildtype IAV infected cells was set 1. N-fold luciferase activity of IAV-infected cell extracts is shown on a logarithmic scale. CMV-luc: CMV promoter-driven luciferase reporter; NS1 variants see above; *p* < 0.05 compared to NS1 184-188. The experiment was repeated four times.

We have previously shown that transfection of cells with an NS1 lacking its two NLS is able to inhibit host gene expression as effectively as wild-type NS1 when a synthetic NLS relocates it to specific speckle-like structures of the nucleus^4^. To prove that this holds also in the course of an infection, we rescued the corresponding recombinant virus by reverse genetics (Fig. 1A). To visualize the NS1 protein, we used the split-GFP system^25^. We fused the GFP-11 domain to the NS1 C-terminus and transduced recipient cells with the pQCXIP-GFP1-10 plasmid. In this first experiment we compared subcellular distribution of NS1-GFP11wt, NS1 38,41 (R38A, K41A, main native NLS mutated), NS1 38…220-GFP11 (R38A, K41A, K219A, R220A, both native NLS mutated), and NLS NS1 38…220-GFP11 (SV40-NLS containing NS1 38…220) upon infection (Fig. 1B).

Infection of cells expressing GFP1-10 demonstrated that the GFP11-containing versions of wild-type (wt) NS1 or NS1 38,41 are diffusely distributed in the nucleoplasm and—in minor amounts—in the cytoplasm. Additionally, both seem to be absent from nucleoli. In contrast, infection with IAV coding NLS-NS1 38..220 or NS1 38..220 shows an accumulation of NS1 in granular structures of the nucleus (Fig. 1B). To investigate whether the mutated IAVs are able to inhibit host cell transcription, cells were infected with the recombinant IAV and were immediately after infection incubated in medium supplemented with a transfection mix containing a luciferase reporter construct. As a control, a virus was used that encodes the mutant NS1 184-188 (GLEWN184-188 RFKRY), known to be unable to bind CPSF30 and to be deficient in transcriptional deregulation. As expected, after the infection with all viruses, the luciferase activity was reduced (by 50 to 90 percent) relative to the control virus encoding the mutant effector domain (NS1 184-188). Also, infection with IAV-NLS-NS1 38..220 strongly attenuated the reporter gene activity compared to the control plasmid (Fig. 1C). Based on these initial observations and given that nuclear speckles are known to play a prominent role in the regulation of gene expression, we asked whether NS1 interferes with the function of nuclear speckles to attenuate the host gene expression.

### NS1 associates with nuclear speckles

**Fig. 2:**
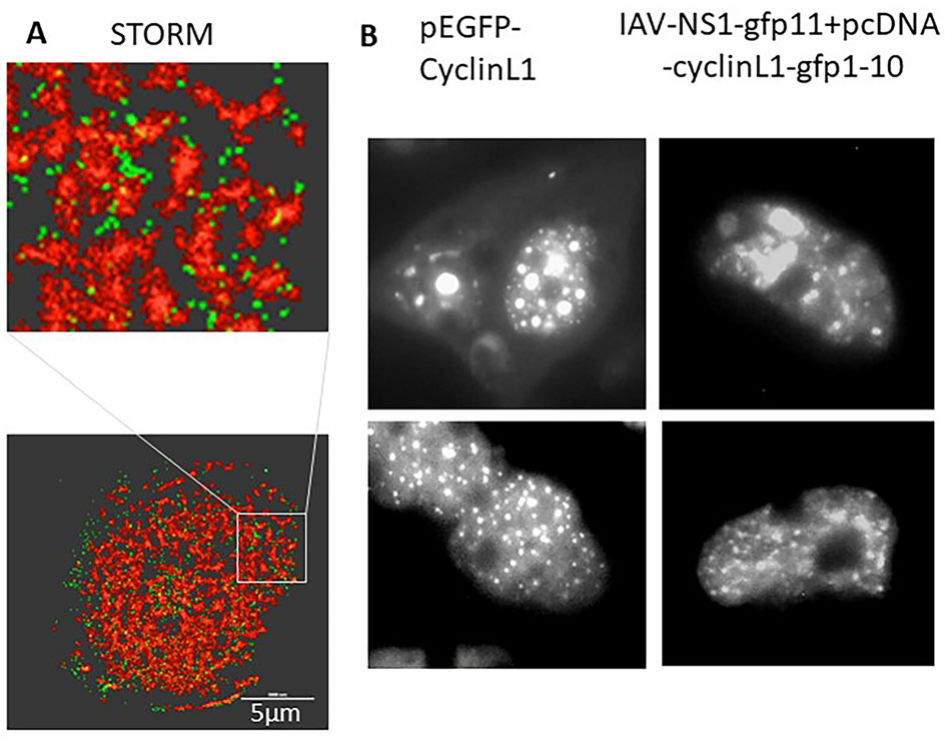
Upon IAV infection wild type IAV-NS1 associates with nuclear speckles. **A** STORM microscopy: cells were incubated overnight with ethynyl-deoxy-uridine and then infected with IAV. 5 h post infection, cells were fixed and a fluorophore was added to the ethynyl moiety by click-chemistry to label cellular DNA (red). NS1 is shown by immunohistochemical staining in green. NS1 can be detected in the interchromatin space. **B**: Left panels show two replicates of cells that were transfected with pEGFP-CyclinL1 as a nuclear speckle marker protein. Cyclin L1 is known to be an immobile component of NSP. The right panels show two replicates of cells that were first transfected with pcDNA3 encoding a protein consisting of the nuclear speckle localising domain of Cyclin L1 (aa318-525) fused to superfolder GFP1-10 and consequently infected with IAV encoding a NS1-GFP11. The pattern of the GFP-induced fluorescent signals is identical or similar between the control cells (pEGFP-CyclinL1) and pcDNA-L1-GFP1-10 transfected and the IAV-NS1-GFP11 infected cells.

Wild-type NS1 shows a diffuse distribution in the nucleus of infected cells. STORM microscopy confirms that NS1 does not co-localize with nuclear DNA. Rather, NS1 seems to be floating in the interchromatin space of the nucleus (Fig. 2A). It has been shown previously that the NSP-specific marker protein SC35 co-localizes with NS1^4^. To further confirm the finding of whether wild-type NS1 specifically co-localizes and/or associates with NSP or interchromatin granule clusters, we transfected cells with a construct encoding the nuclear speckle localizing domain of Cyclin L1, which is known to be an immobile component of NSP that has been fused with GFP 1-10 (green fluorescence protein domain 1-10). These transfected cells were then infected with IAV NS1-GFP11. At the sites of co-expression, both GFP parts reassemble into the fluorescent protein. Figure 2B (right panels) shows that indeed we observe a pattern of fluorescent signals that is identical or very similar to that of pEGFP-Cyclin L1 transfected control cells, demonstrating that NS1 associates with NSP in the course of a genuine infection.

### NS1 that is spatially constrained to NSP inhibits reporter gene expression

**Fig. 3:**
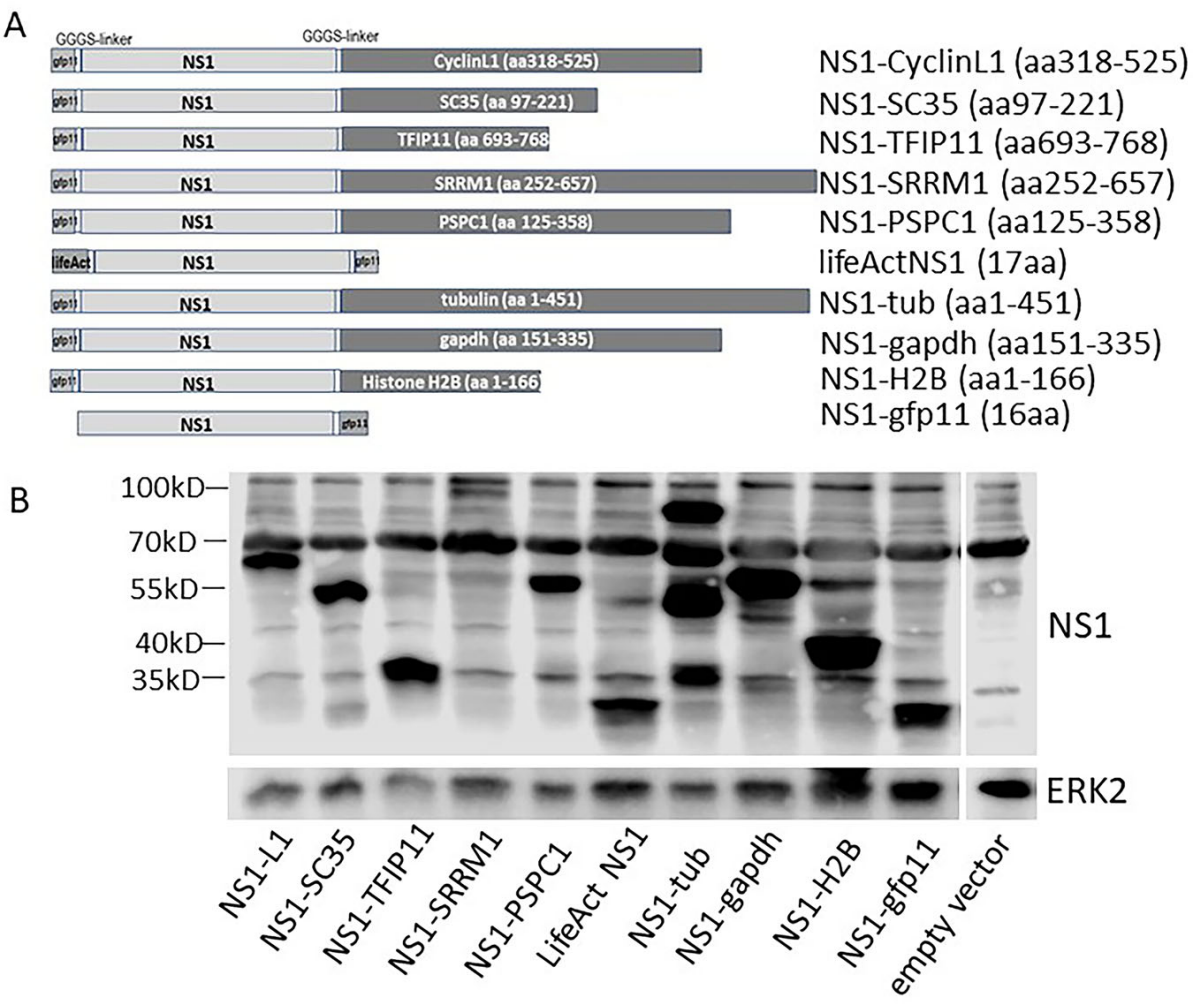
NS1 fusion constructs. **A** Schematic representation of the NS1 constructs used to guide NS1 to different subcellular sites. **B** Western blot to prove expression of the constructs described above. GFP11: the 11th domain of GFP superfolder protein; aa: amino acid, numbers describe the sequence of the indicated protein that was fused to NS1; H2B: histone 2B; SRRM1: human serine and arginine repetitive matrix protein 1; SC35: human Serine/arginine-rich splicing factor SC35; PSPC1: human paraspeckle component 1; TFIP11: human tuftelin interacting p;rotein 11; GAPDH: calatytic domain of GAPDH; NS1-gfp11wt: wild type NS1 fused with GFP11; lifeAct: yeast lifeAct peptide that mediates binding to actin filaments; GGGS: (Gly)3Ser-peptide linker to enable proper secondary structure formation of the domains; aa: amino acid position. Each NS1 in these constructs is mutated at positions 38 and 41 (R38A, K41A) to inactivate the internal nuclear localisation site.

**Fig. 4:**
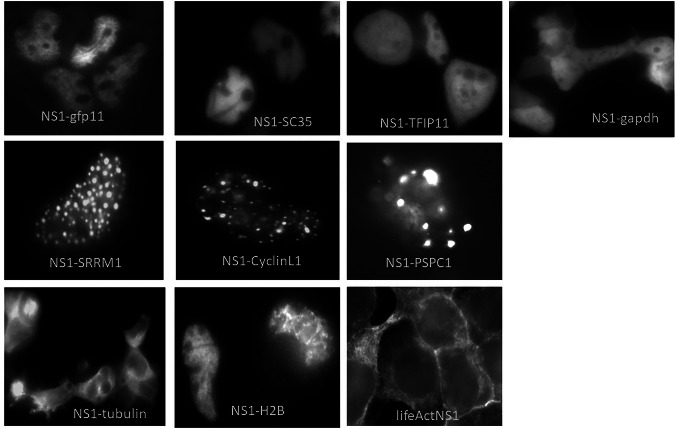
Fluorescence microscopical analysis shows the subcellular localisations of NS1 fusion-proteins (as described in Fig. 2). NS1 fusion proteins were visualised in vivo using the split-GFP assay. SRRM1: Nuclear speckle localising domain (NSLD) of human serine and arginine repetitive matrix protein 1; SC35: NSLD of human Serine/arginine-rich splicing factor SC35; PSPC1: NSLD of human paraspeckle component 1; lifeAct: yeast lifeAct peptide that mediates binding to actin filaments; 2HB: histone 2B; TFIP11: NSLD of human tuftelin interacting protein 11. A representative picture is shown for each transfected plasmid.

**Fig. 5:**
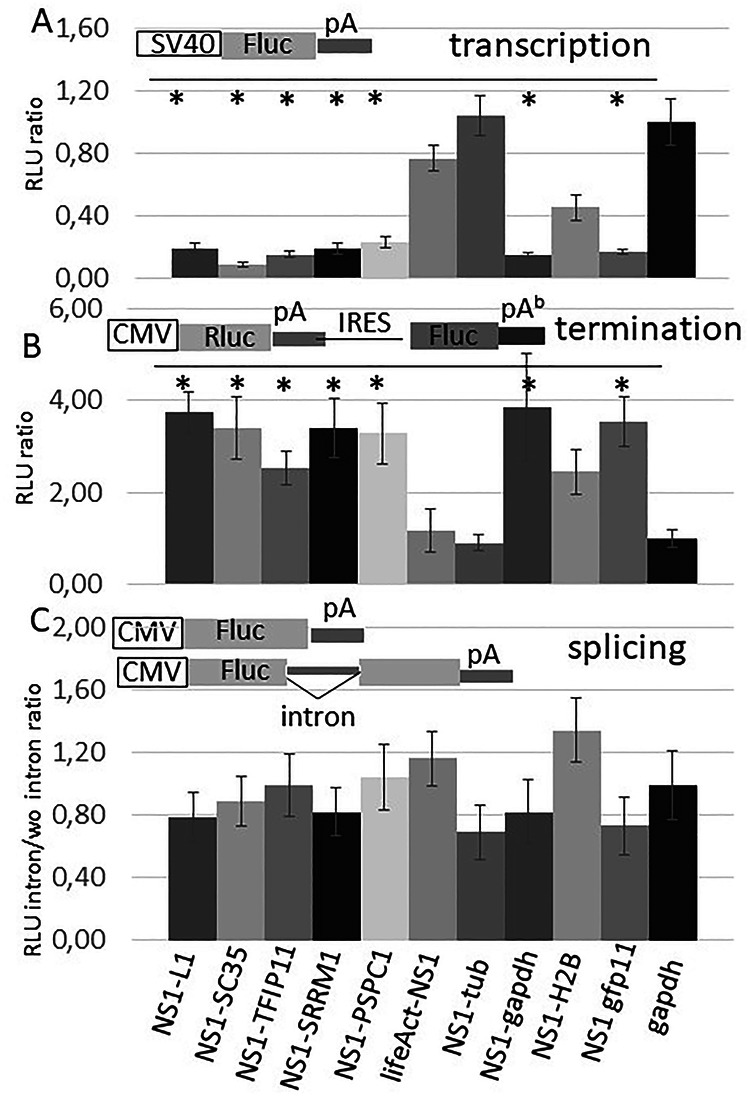
Inhibition of reporter gene activity is dependent on subcellular localisation of NS1. **A** NS1-fusion protein encoding constructs were co-transfected with an SV40 promoter driven reporter gene as indicated. The reporter gene activity measured from extracts of GAPDH (instead if a NS1 fusion) transfected cells was arbitrarily set to 1. **B** same experiment as in **A**, however, cells were co-transfected with a bi-cistronic construct as indicated to measure transcriptional run through of the Renilla-luciferase (Rluc) poly A termination site (construct described in ref. ^4^). The reporter gene activity measured from control extracts of GAPDH (instead of a NS1 fusion) transfected cells was arbitrarily set to 1. **C** NS1-fusion protein encoding plasmids were co-transfected either with an intron-containing reporter gene or intron-less reporter gene construct (addgene #62857 and #62858). The ratio of the activities between intron and intron-less extracts of each NS variant was calculated for each NS1 encoding construct and for GAPDH control. Again, the reporter gene activity measured from extracts of GAPDH (here the ratio with intron/intron-less) (instead of an NS1 fusion) transfected cells was arbitrarily set 1. NS1 constructs see legend of Fig. 2; pA poly adenylation site, Fluc firefly luciferase, Rluc Renilla luciferase, CMV CMV promoter; the experiments were repeated five times. *p < 0.05 compared to GAPDH control.

We fused NS1 to protein domains that target it to specific subcellular structures and verified the desired localization of NS1 microscopically using the split-GFP system. We then tested its potential to inhibit host gene expression and dysregulation of host transcriptional poly A signal-mediated termination via reporter gene assays. First, we fused NS1 to domains known to guide the native proteins to NSP. These domains originate from the NSP marker proteins SRRM1 (aa 252–657), SC35 (aa 97–221), TFIP 11 (aa 693–768), and Cyclin L1 (aa318–525) and are reportedly known to guide the protein to NSP (see https://www.uniprot.org). Cyclin L1 is even known as an immobile component of the nuclear speckle compartment^26^. Furthermore, we fused NS1 to the domain of PSPC1 (aa 125–358) that guides PSPC1 to paraspeckles. As controls, we fused NS1 to tubulin and the LifeAct peptide^27^, localizing the corresponding NS1 fusions to cytoskeletal elements. Finally, NS1 was fused to the GAPDH catalytic domain (NS1 distributed in the whole cell) and Histone 2B (NS1 associated with DNA), respectively (Fig. 3A). Since the NS1’s RNA-binding domain is not involved in host gene shutoff and to ensure that its internal NLS does not influence localization of NS1 fusion proteins, all the NS1 variants were mutated to alanine in positions aa 38 and 41 (R38A, K41A), thereby abrogating the corresponding NLS (along with NS1’s RNA-binding properties). To visualize the subcellular localization of the NS1 fusion proteins, in all constructs, the NS1 is additionally fused with a GFP11 domain. Expression of the NS1 variants upon cellular transfection of the corresponding constructs was verified by Western blot analysis (Figs. 3B, S1). All constructs except the NS1-tubulin showed the expected molecular weight. NS1-tubulin was obviously partially degraded or translation was prematurely terminated. However, as seen below (Fig. 4), the subcellular localization suggests that the fused tubulin sequences seem to be sufficient for tubulin binding. To verify that NS1, which has been fused to the proteins/protein domains described above, is guided to the expected subcellular structures, cells stably expressing GFP1-10 were transfected and analysed by fluorescence microscopy. Figure 4 demonstrates that NS1 wild type, NS1-SC35, and NS1-TFIP11 are diffusely distributed in the nucleoplasm. NS1-GAPDH (catalytic domain) is localized in the cytoplasm as well as in the nucleus (Fig. 4, upper row). The middle row of Fig. 4 shows the subnuclear localization of NS1-SRRM1, CyclinL1, and PSPC1, which are all exclusively associated with nuclear or paraspeckles, respectively. The NS1 proteins fused to lifeAct and tubulin show a clearly non-nuclear, cytoskeletal association, as expected. Fusion of NS1 with Histone2B (H2B) leads to a tight association with nuclear DNA/chromatin, demonstrated by the reticular structure (Fig. 4, lower row). To investigate the potential of the various NS1 proteins to inhibit host transcription, the NS1-encoding plasmids were co-transfected with the reporter plasmid pSV40-luc, encoding a luciferase driven by the constitutive SV40 T-Ag promoter. All lysates of cells co-transfected with plasmids encoding NS1 variants fused to domains of various nuclear speckle marker proteins showed a reduced luciferase activity, representing 10–30% of that measured in control cells that were co-transfected with a construct that expressed GAPDH. The inhibition of the SV40 T Ag promoter-driven expression is as strong as the inhibition mediated by NS1 wild-type co-expression (NS1-gfp11). The NS1-GAPDH fusion protein, which is equally distributed in the cytoplasm and nucleoplasm, also attenuates reporter gene activity as NS1 wild type. Co-expression of NS1-PSPC1 fusion, which is associated with paraspeckles, also led to an inhibition of the luciferase expression. In contrast, co-expression of NS1 guided to cytoskeletal structures of the cell, such as actin or tubulin, failed to inhibit luciferase expression. Even NS1, which is associated with nuclear DNA by its fusion with histone 2B, is not able to fully shut down the constitutive promoter that drives the luciferase gene as it is done by wild-type NS1 or nuclear speckle-localized NS1 (Fig. 5A).

### NS1 that is associated with nuclear speckles dysregulates poly A signal mediated transcriptional termination

NS1 has been shown to dysregulate poly A signal-mediated termination of host transcription^1–4^. Thus we further investigated whether the spatial association of NS1 with NSP is also essential for this dysregulation of termination of host transcription. We used a reporter construct that encodes a Renilla luciferase coding sequence fused with a poly-A termination signal followed by an IRES sequence and a firefly luciferase encoding sequence^4^ (Fig. 5B). Transcription that runs through the Renilla luciferase poly A signal results in firefly luciferase activity. Co-transfection of this reporter with the plasmids encoding NS1 that associates with cytoskeletal structures leads to a low luciferase activity, as does co-transfection of a GAPDH-encoding control plasmid, suggesting that in these cells the Renilla luciferase poly A signal leads to a proper transcriptional termination. However, co-transfections of those plasmids encoding NS1 proteins that are guided to NSPs or paraspeckles resulted in a high firefly luciferase activity similar to the co-transfection of an NS1 wild-type encoding plasmid (Fig. 5B), suggesting that the presence of NS1 in NSP causes run-through transcription due to a failure in poly-A-mediated transcriptional termination.

### Splicing is not affected by NS1 residing in NSP

Since NSPs are reportedly highly enriched in mRNA splicing factors, we investigated whether the presence of NS1 in NS affects the splicing of a reporter gene. A luciferase reporter gene with or without an intron was co-transfected with NS1-fusion protein-encoding expression plasmids as described above (Fig. 4). We could not detect a significant difference between the ratios of the luciferase activity of cells transfected with and without intron, irrespective of whether the NS1 is localized to the NSP or is associated with cytoskeletal proteins, suggesting that splicing of reporter gene mRNA is not affected by the presence or absence of NS1 in NSP (Fig. 5C). However, a reporter gene-based analysis alone may not be sufficient to rule out an effect on host mRNA splicing.

### The N-terminal RNA binding domain of NS1 is dispensable for the IAV induced host transcriptional inhibition in nuclear speckles

**Fig. 6:**
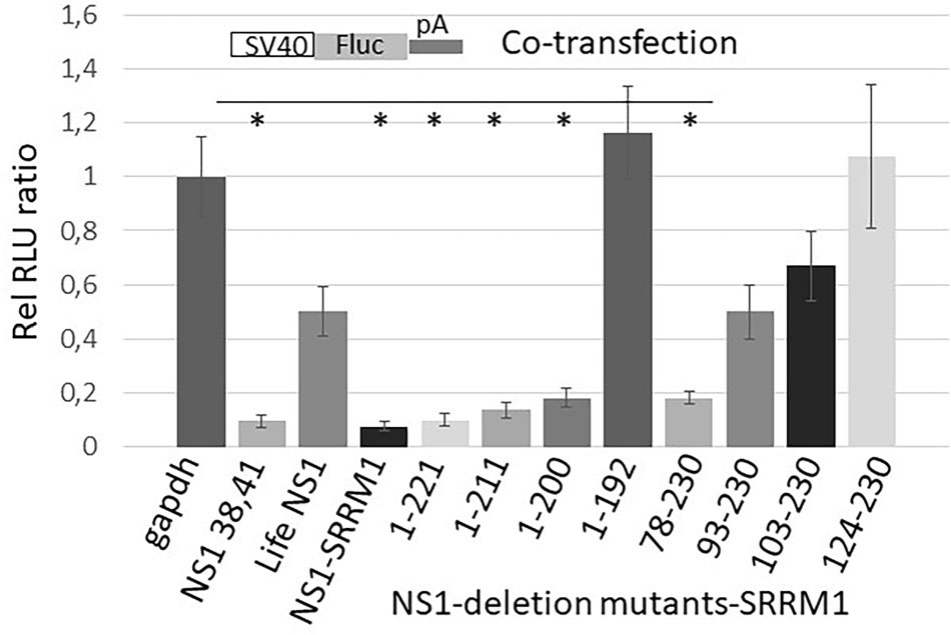
Expression of the NS1 effector domain is sufficient for the deregulation of nuclear speckle-localised reporter gene expression. NS1 N-terminal and C-terminal deletion mutants reveal that the region between amino acids (aa) 78 and 200 is sufficient for NS1-mediated transcriptional inhibition when it is guided to NSP. The number indicates the aa region of NS1 that was fused to the speckle localising domain of SRRM1 and co-transfected with a reporter gene construct. The reporter gene activity measured from extracts of GAPDH (instead of a NS1 fusion) transfected cells were arbitrarily set 1. The experiments were repeated five times. *p < 0.05 compared to GAPDH control

In earlier studies with influenza B virus (IBV), the N-terminal RNA binding domain of NS1 of IBV was shown to be essential for nuclear speckle localization^28^. However, we recently showed that the C-terminal effector domain is responsible for the host gene shut-off effects described here^4,29^. To verify that the results observed with nuclear speckle localized NS1 are mediated by the effector domain of NS1, we constructed N- and C-terminal deletion mutants of NS1 and fused them to the SRRM1 speckle localizing domain (aa 252–657) (Fig. 3). These constructs were co-transfected with an SV40 promoter-driven reporter gene. As shown in Fig. 6, we could demonstrate that N- or C-terminal truncations that remove parts of NS1’s structural elements (beyond residue 78 or before residue 200) considerably reduce its inhibition activity.

### In a genuine infection scenario, the host transcriptional inhibition and deregulation are dependent on the presence of NS1 in nuclear speckles

**Fig. 7:**
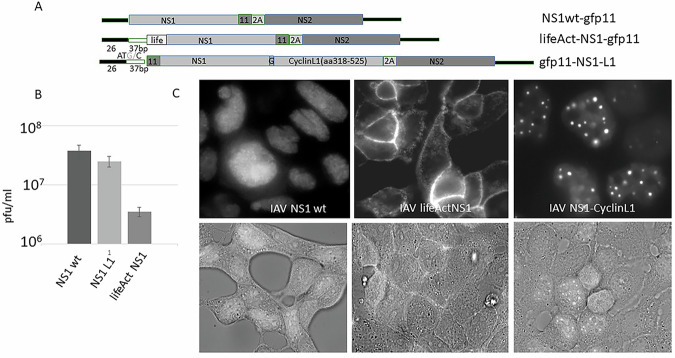
Replication of a recombinant IAV encoding lifeAct-NS1 is attenuated by factor 10 compared to a virus encoding NS1 that is exclusively localised in nuclear speckles. **A** Schematic representation of the pHW2000-NS segment constructs that encode lifeAct-NS1 and NS1-CyclinL1 (NS1-L1); lifeAct-NS1: IAV encoding lifeAct-NS1, lifeAct peptide of yeast N-terminally fused to NS1; ntr: non-translated region; ATG/C: the first ATG was mutated to ATC to elongate the ntr, which was necessary to enhance virus titre; 37 bp: length in base pairs of the sequence homologous to wild type NS segment used to elongate the ntr; 11: the 11th domain of GFP superfolder protein; life: yeast lifeAct peptide that mediates binding to actin filaments; 2 A: 2 A peptide from porcine teschovirus-1; NS1-L1: NS1 CyclinL1 (aa318-525), nuclear speckle localising domain of Cyclin L1; G: (Gly)3Ser peptide; NS1wt: wildtype NS1 C-terminally fused with a gfp11 domain **B** Titration of virus supernatants by plaque assay. pfu plaque-forming unit, the experiments were repeated four times. *p < 0.05 (difference between IAV LifeAct-NS1 and IAV NS1-L1). **C** MDCK cells stably expressing GFP 1-10 were infected with IAVs encoding recombinant NS1 as indicated. In the presence of NS1, self-assembly between GFP1-10 and the 11th domain of GFP fused to the corresponding NS1 protein (split GFP assay). Shown is the analysis of the subcellular localisation of recombinant NS1 upon infection by fluorescence microscopy (upper row) and by an overlay of phase contrast and fluorescence microscopy (lower row).

In the upper experiments, NS1 was ectopically expressed in cells to study host transcription in the absence of any other virus-induced proteins or signals. Upon infection, viral proteins other than NS1 are also involved in the viral takeover of the host metabolism. To confirm the results obtained so far in the context of a genuine viral infection, we constructed recombinant viruses encoding a lifeAct-NS1-GFP11 (actin-binding NS1, lifeAct-NS1) and a GFP-11-NS1-CyclinL1 (NS-associated NS1, NS1-L1). As a control, IAV was used that encodes NS1 wild type (representing full transcriptional inhibition) and NS1 38,41 (R38A, K41A) (demonstrating that full transcriptional inhibition is independent of a functional RNA binding domain) (Fig. 7A). Titration of virus supernatants revealed that the virus encoding lifeAct is attenuated, resulting in a roughly tenfold reduction of viral replication, suggesting that the presence of NS1 in the nucleus promotes replication of IAV (Fig. 7B). The NS1-CyclinL1 encoding IAV (NS1-L1) replicates to titers similar to the control NS1-expressing virus IAV NS1 38,41. The expression level of the NS1 variants during infection was analyzed by Western blot experiments (Fig. S1C). To visualize the subcellular localization of NS1 by the split-GFP system, GFP1-10-expressing cells were infected with the recombinant IAV as described above and microscopically analyzed. Figure 7C demonstrates that lifeAct-NS1, as well as NS1-CyclinL1 expressed after infection with the corresponding IAVs are associated with their expected cellular destinations, as has been observed in the above-described transfection experiments.

Furthermore, an IAV was generated that expresses an NS1 whose linker sequence between the RNA binding domain and the effector domain was elongated by inserting a spacer peptide, in this case the peptide cleavage site PGDEMEECSQHLPG of Hepatitis C Virus NS3 protease flanked by a proline-glycine dimer (NS1 78HCV) (Fig. S1A). The elongated linker resulted in an NS1 that is equally distributed in the cell cytoplasm and nucleus (Fig. S2), thus mimicking the NS1-GAPDH fusion (Fig. 4, upper panel right) used for the transfection experiments described above. Additionally, to further confirm the finding that the RNA binding domain is dispensable for the inhibition of gene expression, an IAV encoding an NS1 effector domain only consisting of aa1-18-GFP11-aa78-230 (NS1-ED) was generated (Fig. S1A). To ensure that the internal NLS does not influence localization of NS1, in all these viruses, the NS1 encoding sequences were mutated to alanine in positions aa 38 and 41 (R38A, K41A).

Whereas the NS1 78 (HCV) is distributed over the whole cell, similar to the NS1-GAPDH fusion protein described above, the NS1-ED is mainly localized in the nucleus (Fig. S2).

**Fig. 8:**
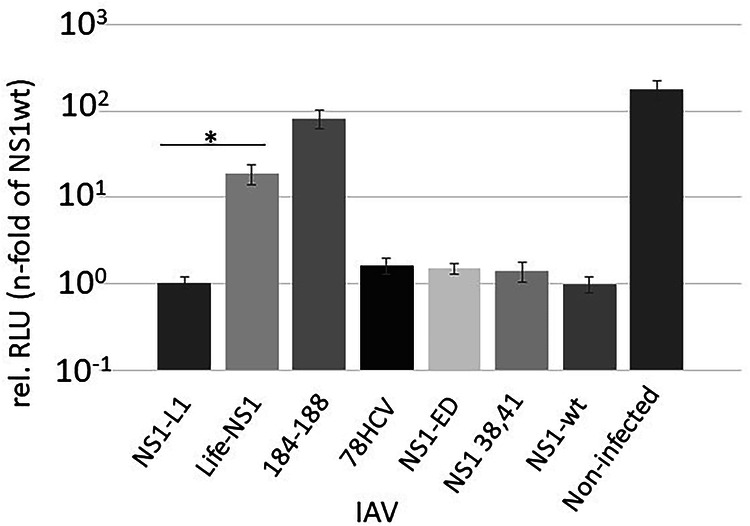
In a genuine infection scenario the presence of NS1 in nuclear speckles is essential for the inhibition of reporter gene expression. Cells were infected with recombinant IAV as indicated and subsequently immediately transfected with a CMV promoter-driven luciferase construct. The luciferase activity from an extract-harvested 6 h p.i. - of an IAV-NS1 wild type (NS1-wt) infected cell was arbitrarily set to 1. Recombinant IAVs are described in Fig. 7 and S1. IAV-NS1-L1 (IAV encoding NS1 fused with Cyclin L1 aa318-525) suppresses the reporter gene significantly stronger than IAV-Life-NS1 (IAV encoding NS1 fused with lifeAct peptide). IAV 184-188: IAV encoding mutant NS1 184-188(GLEWN184-188 RFKRY) is shown as control; IAV 78HCV and IAV NS1-ED (effector domain) see Fig. S1; IAV NS1 38,41: IAV encoding mutant NS1 38,41 (R38A, K41A). All NS1s are additionally fused with GFP11 as described in Fig. S1. The experiment was repeated three times. * P Value < 0.05

To investigate whether the inhibition of host cell transcription in a native infection scenario is dependent on the presence of NS1 in NSP, cells were infected with IAV and then transfected with a luciferase reporter construct. The analysis of the luciferase activity revealed that the nuclear speckle resident NS-CyclinL1 encoding IAV inhibits host transcription similar to wild-type NS1 IAV, whereas the cytoskeletal-associated lifeAct-NS1 encoding IAV failed to attenuate luciferase expression, as the mutant NS1 184-188 encoding virus served as a control, as an effector domain non-functional in reporter gene inhibition (Fig. 8). Furthermore, infection of cells with the IAV encoding NS1-ED (effector domain) as well as NS1 78HCV (Fig. S1A) also resulted in suppression of reporter gene activity (Fig. 8).

### NS1 association with nuclear speckles seems to deregulate poly A-mediated termination of transcription at some gene loci

**Fig. 9:**
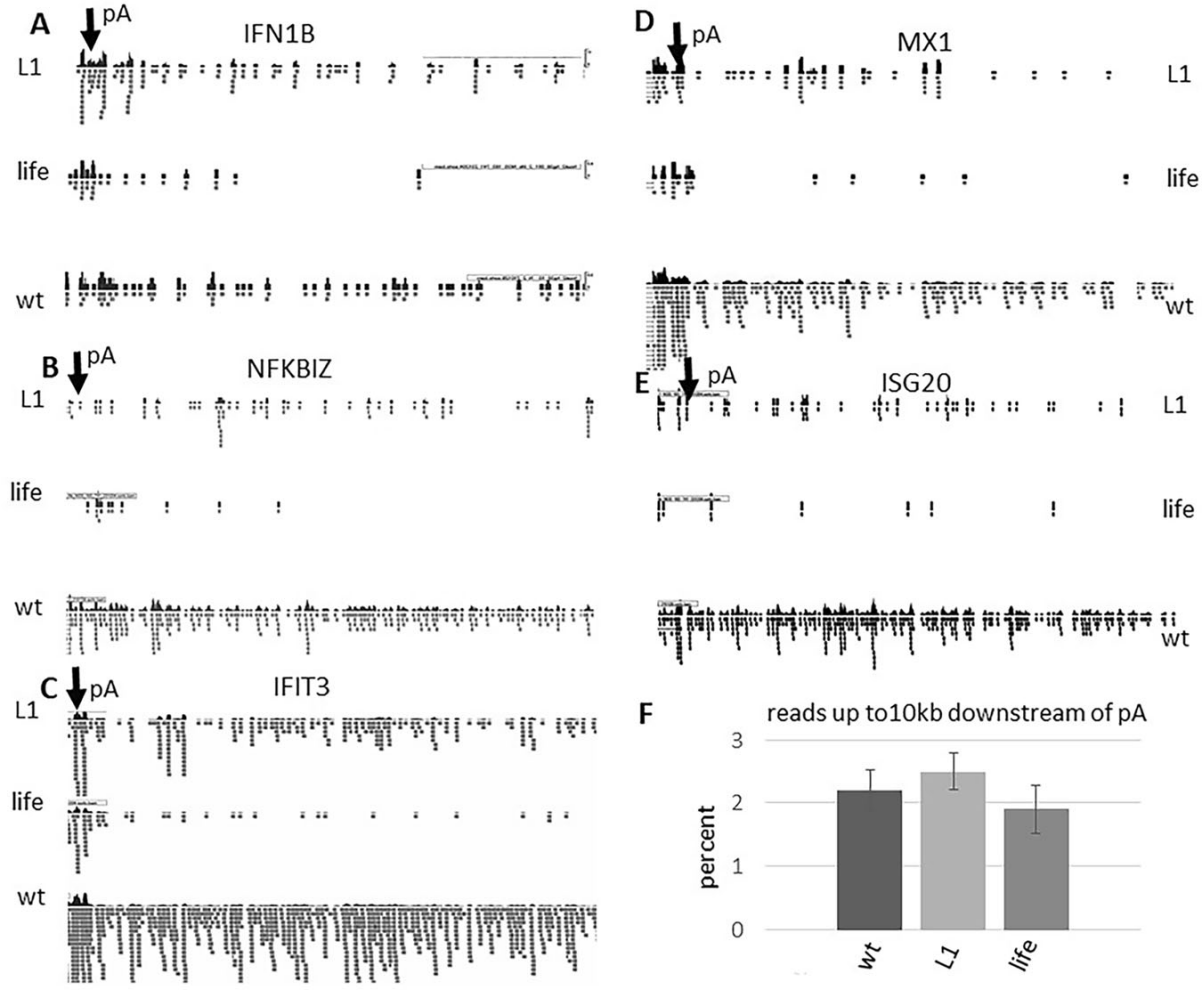
IAV induced deregulation of host gene transcriptional termination affected by the presence of NS1 in nuclear speckles. (**A**–**E**) The transcriptome (6 h p.i.) of IAV infected cells were NGS-sequenced and the sorted reads were aligned to the human genome (GRCh37/hg19). The aligned reads were viewed using the IGV (integrated genome viewer) software from the Broad Institute. Shown are the alignments of the reads downstream of the polyadenylation site (pA=poly adenylation site, marked by an arrow) of the host genes **A** IFN1B (interferon beta 1), **B** NFKBIZ (NFKB Inhibitor Zeta) and **C** IFIT3 (Interferon Induced Protein with Tetratricopeptide Repeats 3), **D** MX1 (Interferon-Induced GTP-Binding Protein Mx1), **E** ISG20 (interferon stimulated gene 20),. Shown is a region of roughly 20 kb downstream of the poly adenylation site of each gene. L1: reads from IAV-NS1-Cyclin L1 (aa318-525) infected cells; life: reads from IAV-lifeAct NS1 infected cells; wt: reads from IAV wt infected cells. **F** Overall the percentage of reads up to 10 kb downstream of poly A sites is not significantly different between wt-IAV, IAV-NS1-CyclinL1, and IAV-lifeActNS1 infected cells suggesting that only a subset of transcriptional terminations is deregulated.

A major feature of IAV infection is the deregulation of host gene poly A-mediated termination of transcription, resulting in DOG transcription. To investigate whether this IAV infection-induced transcriptional dysregulation correlates with the localization of NS1 in NSP, we infected A549 cells with IAV wild type, IAV lifeAct-NS1, and IAV-NS1-CyclinL1. The transcriptomes were sequenced via RNA-Seq NGS (next-generation sequencing), and the reads were aligned to the human genome to analyze the downstream transcription beyond the poly A site of host genes. We observed that the DOG transcription is decreased in IAV-lifeAct NS1-infected cells compared to IAV-CyclinL-infected cells. However, the DOG transcription in IAV-NS1-wildtype-infected cells is even stronger than in the IAV-NS1-CyclinL-infected cells (Fig. 9A–E). We conclude that the NSP-associated NS1-cyclinL correlates in quality, but not in quantity, with the NS1 wt-mediated dysregulation of host gene transcriptional termination, further confirming that the presence of NS1 in NSP mediates the dysregulation of host gene expression. However, as observed earlier by us and others^1–4,29^, the regulation of poly-A termination of some genes (such as histones, GAPDH, and others, data not shown) seems not to be affected by the infection with mutant or wild-type IAV. Moreover, on the whole, the proportion of reads up to 10 kb downstream of poly A signals is not significantly different between lifeAct-NS1, NS1-cyclinL1, and wt NS1 encoding IAV-infected cells (Fig. 9F).

### The NS1-mediated suppression of reporter gene expression is independent of whether the genes are transfected into the cell or are stably integrated into the host cell genome

**Fig. 10:**
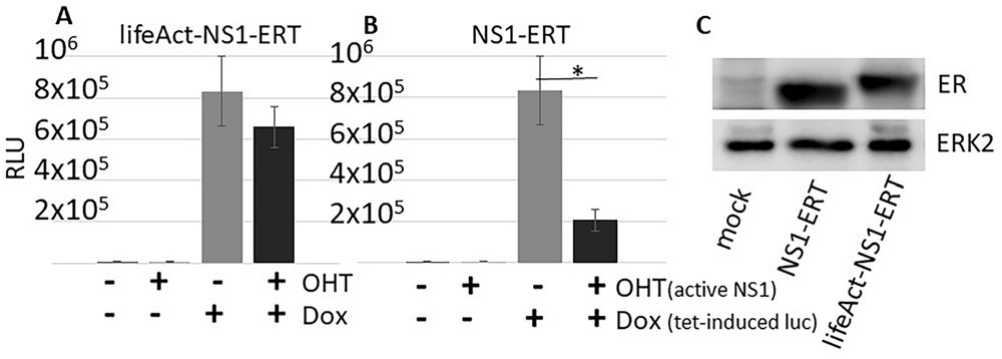
The NS1-mediated suppression of reporter gene expression is independent of whether the genes are transfected into the cell or are stably integrated into the host cell genome. **A** luciferases assay is shown. The HEK293 cells contain a stably integrated, inducible luciferase gene and additionally either a stably integrated, tamoxifen-inducible lifeAct-NS1 or a stably integrated, tamoxifen-inducible wild type NS1 **B**. NS1 suppresses reporter gene expression when both NS1 and reporter gene are stably integrated into the host cell genome. The experiment was repeated threefold; * P < 0.05. OHT: 4-hydroxy-tamoxifen; Dox: doxycycline; ERT: mutant estrogen receptor domain ERT2; RLU: relative light units. **C** Western blot demonstrating the expression of the recombinant NS1-ERT proteins in stably transduced HEK293 cells. ER: antibody specific for estrogen receptor domain, detecting ERT2 domain; NS1: NS1 antibody.

NSP are areas that reportedly can enhance gene expression depending on the dynamics between gene positioning, genome sub-compartments, and gene activity^8^. It was suggested that around 50% of transcriptionally active genes are associated with NSP^30^. However, it is unclear why certain genes are associated with speckles while others are not. It has been shown that the proximity of genes to NSP correlates with high gene expression and vice versa, suggesting that there is a link between nuclear speckles and regulation of gene expression^18,19^. The NS1-mediated inhibition of expression has so far been shown by transfection of a reporter gene located on a small plasmid that may freely “float” through the nucleus and that may thereby be able to associate with NSP. Thus, we investigated whether this observation also holds true for a reporter gene that is stably integrated into the host genome (as host genes are). We used HEK-NS1ERT cells that have been described earlier^29^. They stably express an NS1, which is fused to the mutated 4-OH-tamoxifen (OHT)-inducible estrogen receptor domain ERT2 (HEK-NS1ERT). We further generated cells that stably express a lifeAct-NS1 fused to the ERT2 domain. Both cell lines were additionally retrovirally transduced with a doxycycline-inducible luciferase reporter gene (HEK-NS1ERT-luc/HEK-lifeActNS1-luc). To exclude any effects of retroviral-specific affinities to genome integration sites, we alternatively transferred a doxycycline (dox)-inducible luciferase into HEK-NS1ERT cells using a Sleeping Beauty transposase system to generate HEK293 NSERT-rtTAluc. However, we did not observe any difference between the two cell lines. In HEK-NS1ERT-luc and -rtTAluc cells, luciferase can be switched on by doxycycline, and NS1 can be activated by OHT. Using these cell lines, we could demonstrate that NS1 mediates suppression of a genome-integrated reporter gene (Fig. 10A). The actin-binding lifeAct-NS1-ERT was not able to suppress reporter gene activity (Fig. 10B), confirming the results described above. We conclude that the NS-mediated suppression of reporter gene activity is not a unique phenomenon of transfected reporter genes. We have to keep in mind that the cells used may contain multiple copies of the reporter gene in their genomes. Gene positioning effects may become more relevant if a single integrated copy of a reporter gene were to be integrated.

### The full potential of NS1 to inhibit reporter gene expression correlates with functional nuclear speckles

**Fig. 11:**
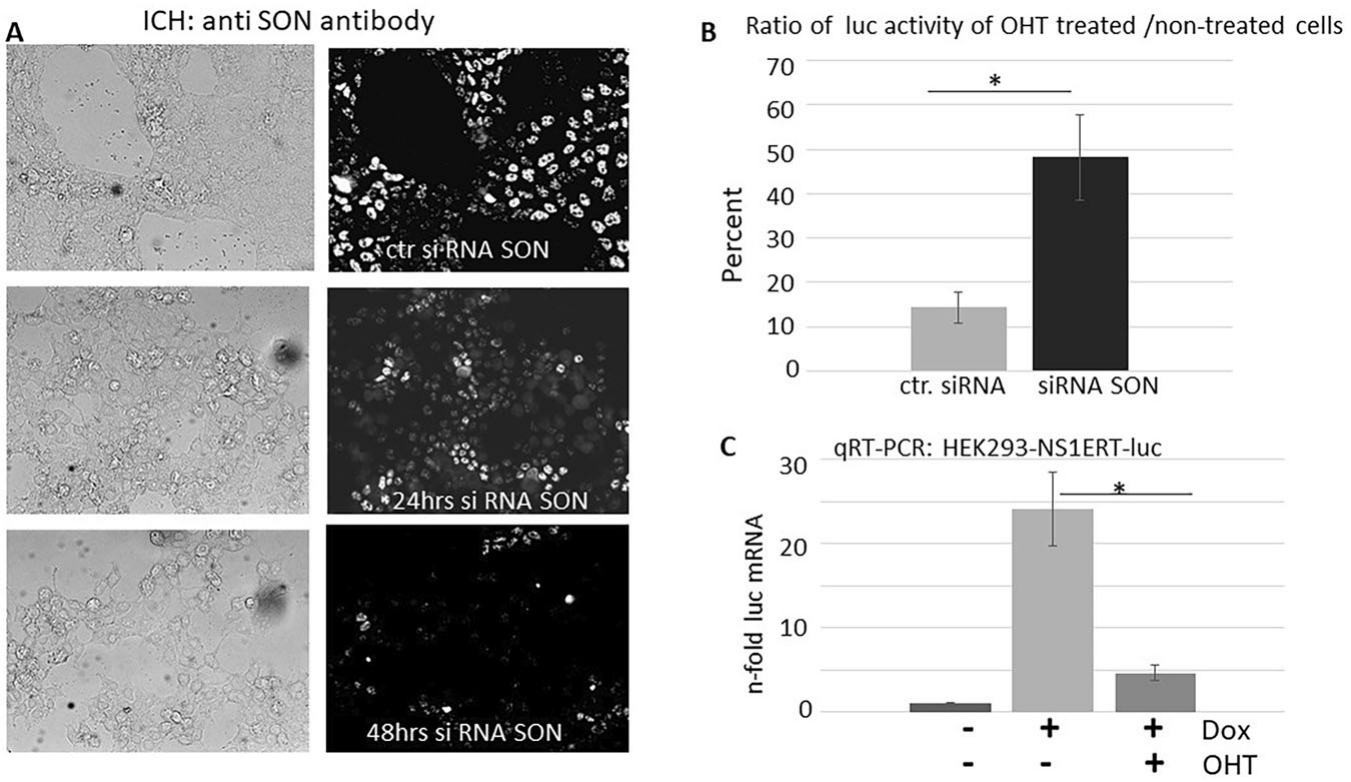
The potential to suppress transcription of a reporter gene by NS1 is higher in cells with functional NS. **A** siRNA targeting SON—an essential protein for functional NSP—was transfected into cells. Depletion of SON was verified by immunocytochemistry using an anti-SON antibody. **B** HEK-NS1ERT-luc cells stably express a 4-OH-tamoxifen activatable NS1 (NS1 fused with a tamoxifen inducible estrogen receptor domain)^29^ and a doxycyclin inducible luciferase. Cells were transfected with siRNA SON or with control siRNA. Luciferase expression was induced more than 40 h post transfection. The percentage of luciferase activity from active NS1-containing cells with/without SON depletion is shown compared to the activity of cells containing inactive NS1 ( = set 100 percent). The experiment was repeated threefold; *P < 0.05 **C** qRT-PCR of HEK-NS1ERT-luc cell RNA demonstrates that dox induces luciferase mRNA of a stably genome-integrated luc-gene. The activation of NS1 by OHT suppresses luc transcription. (dox doxycycline, OHT 4-OHT-tamoxifen, ctr control, luc luciferase); the experiment was repeated threefold; *P < 0.05.

We further asked whether functional NSPs are essential for stably chromatin-integrated reporter gene expression. We transfected these HEK-NS1-ERT-luc cells with SON siRNA (Fig. 11A) to knock down SON expression. Since SON is an essential structural component of NSP, depletion of SON, as observed in Fig. 11A after 24 and 48 h of siRNA treatment, leads to cells containing dysfunctional NSP. Luciferase activity was much higher (roughly tenfold) in cells with functional NSP compared to SON-deficient cells, demonstrating the prominent role of NSP for the expression of intron-less reporter genes (data not shown). Then, the potential of NS1 to inhibit the expression of induced reporter gene expression in cells with versus without functional NSP was compared. In the absence of functional NSP (+SON siRNA), the NS1-induced luciferase activity drops to roughly 50 percent when NS1 has been activated by the addition of 4-HO-tamoxifen (+OHT) compared to inactive NS1 (−OHT). In the presence of functional NSP (+ctr siRNA), the luciferase activity drops roughly to 15 percent when functional NS1 is induced by OHT compared to an inactive NS1. The data demonstrate that the potential of NS1 to inhibit the expression of a genome-integrated reporter gene is significantly larger in the presence of functional NSP (Fig. 11B).

### The NS1-mediated inhibition of luciferase activity is due to a reduced transcription

We previously demonstrated by RNA labeling experiments that NS1 leads to a global reduction of cellular transcription^4^. However, NSPs are predominantly described as sites of mRNA maturation and not as sites of transcription. Thus, we wished to specifically investigate the question of whether the here observed NSP-dependent attenuation of a stably integrated luciferase reporter gene by NS1 relies on reduced mRNA transcription. We used the above-described cells that stably express an OHT-inducible NS1ERT and a dox-inducible luciferase. QRT-PCR of total RNA of non-treated/dox-treated and OHT-treated/dox-treated cells revealed that dox induced the luciferase mRNA twenty-fourfold. In the presence of OHT, the luciferase mRNA induction by dox dropped to fourfold (Fig. 11C). Because the GAPDH transcription may be affected by NS1 expression, we calculated the CT values either for 5SRNA, telomerase, or Alu repeat as reference genes. Irrespective of which of the three genes was used as a reference, there were no differences in the relative induction of luciferase transcription. These data confirm that NS1-mediated inhibition of luciferase activity observed here is due to a reduction of transcription.

## Discussion

IAV, unlike most other RNA viruses, replicates in the nucleus, a feature that places evolutionary pressure on a number of viral proteins to cooperate or interfere with host chromatin-based regulatory processes in infected cells^2^. IAV encodes the non-structural protein NS1, which antagonizes host antiviral responses^30,31^. This occurs through multiple mechanisms, including inhibition of virus sensing and suppression of host functions that are detrimental to the virus, such as host transcription and translation^31^. The IAV-NS1 protein is known to specifically inhibit host gene transcription and to deregulate transcriptional termination^2–4^.

We should have in mind that we here used IAV H7N7 SC35M as a model IAV that encodes a CPSF30-binding NS1. We and others described earlier that NS1 from various human and zoonotic IAV strains showed a strong suppression of reporter gene expression, which correlates with the ability of NS1 to bind CPSF30^4,29,32,33^. However, there are also IAV strains, such as IAV H1N1 A/Puerto Rico/8/1934 (PR8), that behave differently, most likely because of a missing or reduced ability of NS1 to bind CPSF30^29^.

Here we present evidence that NS1 associates with NSP, which may result in dysfunctional NSP, which in turn causes transcriptional inhibition/deregulation of host genes. The conclusion is mainly based on the NS1 fusions localizing NS1 to NSP, whereas the fusion with the LifeAct peptide excluded NS1 from the nucleus. The presence of NS1 in NSP specifically mediates reporter gene transcriptional inhibition. Non-nuclear actin-binding LifeAct-NS1 was neither able to inhibit transcription nor to deregulate host transcriptional termination. Thus, we here identified NSP as the organelle that gets—from the host cell’s point of view—dysfunctional by the presence of NS1 in the course of an IAV infection. We further observe that an IAV encoding lifeAct-NS1—in contrast to NS1-CyclinL1—replicates to lower titers than IAV, strengthening the view that the presence of NS1 in NSP may be important for viral replication.

Interestingly, IBBV NS1 has been shown to accumulate in NSP independent of other viral functions. However, in disagreement with our observations, this accumulation seems to be dependent on the N-terminal RNA-binding domain of NS1^28^.

Early observation already indicated a functional and spatial interaction of IAV-NS1 and NSP. In the course of an IAV infection, the normal speckled pattern of the SC35 NSP marker protein was altered to generate a more punctate distribution, suggesting that NS1 may induce changes in nuclear speckle structure and function. These alterations in the nuclear location of small ribonucleoprotein particles were also induced by the sole expression of NS1 protein^34^. Additionally, IAV utilizes NSP to promote post-transcriptional splicing of its M1 mRNA. Since NS mRNA was not enriched at NSP, this may indicate that M mRNA specifically utilizes NSP for RNA processing. Interestingly, the presence of NS1 protein in NSP promotes M1 mRNA splicing and export^35^. Depletion of SON, an essential protein for nuclear speckle formation, reduces IAV replication, further supporting the importance of NSP for IAV replication^36^. However, due to the general importance of this organelle for cell growth, no specific conclusion can be drawn from this finding. Here, we demonstrate that SON depletion reduces the relative potential of NS1 to inhibit reporter gene transcription, suggesting that the full potential of NS1 to suppress host gene expression unfolds in functional NSP. The observation that NS1-expressing cells show growth retardation^29^ as well as siRNA SON-treated cells^36^ is also in line with the hypothesis that NS1 may affect the proper function of NSP.

The exact role of NSP in the nuclear machinery is still under debate. A lot of data support the view that in NSP mRNA splicing and maturation take place^11–15,37^. In our study, however, it is an intron-less reporter gene whose expression is attenuated by NS1. Moreover, we demonstrate that NS1 specifically suppresses the transcription of an intron-less reporter gene. Splicing seems to be unaffected according to our data comparing an intron-containing version with an intron-less version of the reporter gene. However, more investigations are clearly needed to draw final conclusions with respect to splicing in NSP when NS1 is present. It was observed that many endogenous intron-less mRNAs, which do not undergo splicing, associate with nsp. The authors conclude from their data that export-competent messenger RNP assembly occurs in NS for intron-less mRNAs and that entering NSP serves as a quality control step in mRNA export^38^.

Some recent reports focus on the role of NSP in transcription and, specifically, enhancement of transcription^8^. A study using proximity labeling (TSA-seq) determined distances between genes and nuclear speckles. These data revealed a surprisingly conserved and deterministic genome organization pattern^18,39^. The distances were found to be highly predictive of gene expression levels, such that proximity correlates with high gene expression and vice versa, strengthening the idea that there is a link between nuclear speckles and regulation of gene expression^18^. We also observed an inhibition of reporter gene activity when NS1 is associated with paraspeckles instead of NSP. We here did not follow up on this observation to confirm this finding by further experiments. However, it is known that paraspeckles increase upon infection with RNA viruses^40^. Furthermore, an IAV infection induces the expression of the paraspeckle-specific lncRNA NEAT and leads to an excessive formation of paraspeckles^41^. Additionally, we speculate that the residual inhibitory potential we observed in SON-depleted cells may arise from NS1 associated with paraspeckles.

Upon IAV infection, rampant transcription rapidly reorganizes host cell chromatin interactions. Transcriptional run-through by RNA polymerase II seems to remodel genome 3D architecture^42^, and nuclear bodies are known to change structure, number, and also function upon IAV infection. Here, we present evidence that the presence of IAV NS1 in nuclear inter-chromosomal domains may result in functionally misdirected nuclear speckles as part of the viral takeover of the cellular metabolism.

## Materials and methods

### Cells, plasmids, viruses, chemicals and antibodies, siRNAs

Reporter plasmids CMV-LUC2CP/ARE and CMV-LUC2CP/intron/ARE (Gideon Dreyfuss, addgene #62857 and #62858), pQCXIP-GFP1-10 (gift of Yutaka Hata, addgene #68715), and pCW57.1-luc (gift from Stephen Tapscott, addgene #99283), pSBtet rtTA G72V SE Δsplice GP Luc (gift of Alexander McLellan addgene #194328), SB100X in pCAG globin pA (gift of Mark Groudine, addgene #127909) were obtained from Addgene. pSV40-luc. pCMV-luc and pCMV-RlucpA-IRES-FlucpA were described earlier^4^. mCherry2-SRRM1 was used as a source for the nuclear speckle localising domain of SRRM1 and was obtained from Addgene (#54563). pEGFP-Cyclin L1 was used as a source for the Cyclin L1 nuclear speckle localisation domain (gift of W. Becker, Aachen)

HEK293 (transfections), A549 (RNASeq, infections), or MDCK (split gfp assays) cells were used throughout this study. Cells expressing NS1ERT (HEK-NS1ERT, NS1 fused to the OHT-inducible estrogen receptor) have been described earlier^4^. NSERT expressing cells were either transduced with pCW57.1-luc (results in HEK-NS1ERT-luc) or co-transfected with both pSBtet-rtTA-G72V-SE-Δsplice-GP-Luc and SB100X (Sleeping Beauty transposase) in pCAG globin pA to generate HEK293 NSERT-rtTAluc. These cells contain a doxycycline-inducible luciferase gene and a 4-hydroxy-tamoxifen (OHT) inducible NS1ERT.

pQCXIP-GFP1-10 was used to generate GFP1-10 expressing HEK293 or MDCK cells to detect GFP-11 domain containing proteins via the split GFP reassembly assay.

All NS1-coding sequences used in this study originate from the seal influenza virus strain SC35M (H7N7), which is a mouse-adapted virus originally isolated from seals, however, of avian origin. SC35M was chosen as a model of an IAV encoding a CPSF30 binding NS as it is present in many human as well as zoonotic IAVs.

Recombinant SC35M IAV was generated using the 8-plasmid method based on pHW2000 vector^43^. To enable the generation of any mutation in NS1 without interfering with the expression of overlapping NS2 we constructed an NS segment that separates the expression of NS1 and NS2 gene by a 2 A peptide as described earlier^4^.

PCR and cloning of mutant NS1 were performed according to standard methods. Transient overexpression of NS1 was performed by cloning the corresponding ORFs (open reading frames) into pcDNA3 by standard cloning techniques. The expression levels of all expression plasmids have been controlled by western blotting. To visualize NS1-fusion proteins used in fluorescent microscopy, the GFP-11 domain was added, and plasmids were then transfected into cells that were transduced with pQCXIP-GFP1-10, resulting in fluorescence when GFP11 fusion protein and GFP1-10 complement each other by self-assembly (split GFP assay). The sequences of the nuclear speckle localising domains from TFIP11, SRRM1, PSPC1, and SC35 were extracted from “https://www.uniprot.org”. The corresponding sequences were either amplified by standard PCR from parental plasmids or from commercially synthesized DNA strands (Eurofins, Germany).

Commercially available antibodies as pan-ERK2 antibody (Santa Cruz Biotechnology), NS1 (GeneTex, USA) were used to detect proteins analysed by western blotting. Anti-SON antibody (Sino Biological Inc., #207034-T02) was used to detect SON in immunocytochemistry. Commercially available antibodies were used to detect oestrogen receptor ER-alpha (Cloud-clone Corp., USA)

### siRNA experiments and immunocytochemistry

SON siRNA was purchased from Horizon Discovery (siGENOME SMARTpool # M-012983-01-0005). RNA was transfected using DharmaFECT transfection reagent according to the manufacturer’s instructions. Since SON is difficult to detect by western blotting due to its size of 263 kDal, the efficiency of gene suppression was analysed by immunocytochemistry. SiRNA-treated cells were fixed with 4% PFA, subsequently permeabilised with 0,2% Triton and blocked with 10% serum. SON was detected using anti-SON Antibody (Sino Biological Inc., #207034-T02) and a secondary fluorescently tagged anti-rabbit antibody.

### Fluorescence and STORM microscopy

Fluorescence was performed as described previously^44^. Briefly, cells expressing GFP1-10 were seeded in 15 µ-slide chambers (ibidi GmbH, Germany) and were either transfected with a GFP11-NS1 expressing plasmid or infected with an IAV encoding a GFP11-NS1 protein. NS1 was visualised using a laser scanning microscope (LSM 780, Zeiss, Oberkochen, Germany) equipped with a 100x, **×**63 (NA 1.4) oil immersion or ×40 Plan-Apochromat objectives (Zeiss, Oberkochen, Germany). All images were analysed with the program Fiji (ImageJ).

STORM microscopy was performed as described^4,32^. Briefly, cells were seeded into cell culture wells and treated with ethynyl-deoxy-uridine (EdU, 1 μM, over night). Next day, they were infected with recombinant IAV (encoding a his tagged NS1)^44^. 5 h post infection, cells were fixed and a fluorophore was added to the ethynyl moiety by click-chemistry to label cellular DNA according to the manufacturer’s protocol (Jena Bioscience). Then fixed cells were immunostained with an anti-his-tag antibody (ThermoFisher, Germany) and a secondary fluorescent antibody, and applied to STORM microscopy. STORM sample preparation was done according to the reporter-only method with conventional antibodies according to the Nikon N-STORM protocol for immune staining. Imaging of the samples was done with the N-STORM Ti-LAPP Ti laser application system. The calculation of the image reconstruction was done with NIS-Element Advanced Research – Imaging software (V4.51.01) using algorithms for molecule identification and drift correction. Fluorescence microscopy was performed according to standard methods. Cells were analysed using a Zeiss Confocal LSM800 microscope using the Zen Lite system with the Plan-Apochromat 40**×**/1.40 Oil DIC M27 objective and AiryScan GaAsP detector (1AU). Pictures were prepared with Fiji/ImageJ software version 1.51n.

### Luciferase assay

Luciferase assays were performed as described earlier^4^. The RLU (relative light units) values were set in relation to RLU values of GAPDH transfected cells (was set 1).

### Statistics

Statistical significance was evaluated by Student’s *t* test (*P < 0.05). Generally, the error bars represent the standard deviations or 95% confidence interval, respectively.

### SDS PAGE and western blotting

SDS polyacrylamide gel electrophoresis and western blotting have been described earlier^4^.

### RNA isolation and qRT-PCR

Total RNA was isolated using the MONARCH kit (NEB, USA) following the manufacturer’s protocol. Subsequently, the RNA was reverse transcribed into DNA following the standard procedures. cDNA was used to perform qRT-PCR using a SYBR Green master mix (Thermo Fisher) and a Roche LightCycler 480-II. As reference the RNA of the telomerase (fwd: 3’-tgtgcaccaacatctacaagatcc-5’; rev: 5’-ctgatgaaatgggagctgacg-3’), alu repeat (fwd: 5’-acgcctgtaatcccagcactt-3’; rev: 5’-tcgcccaggctgggtgca-3’) and 5SRNA (fwd: 5’-ggccataccaccctgaacgc-3’; rev: 5’-cagcacccggtattcccagg-3’) encoding cDNA was used for normalisation to calculate the n-fold values according the delta-delta ct method^33^. Irrespectively, which of the three genes was used as a reference, there were no differences in relative induction of luciferase transcription (fwd: 5’-aggcccggcgccattctatc-3’; rev: atttgtattcagcccatatcgttt-3’). The difference of the ct-values between treated and non-treated cells was calculated (∆ct) and the n-fold (2∆∆ct) difference is shown^45^. The non-treated cells were set to 1. The experiment was repeated three times.

### RNA-seq by NGS

A549 cells were infected with wild-type and mutant IAV as indicated (multiplicity of infection [MOI] of 3). Six hours post-infection (p.i.), total RNA was isolated. The library preparation of the total RNA (depleted for rRNA) was performed with the NEBNext Ultra II RNA directional kit, and single-read sequencing was performed using a NextSeq 500 System with a read length of 75 bp. Using a molecular barcode, the samples have been demultiplexed (bcl2fastq2) to fastq data and quality controlled (FastQC). Trimmomatic has been used for adapter trimming and read filtering. The resulting reads were aligned to the Ensembl GRCh38 reference genome using Hisat22. The aligned reads were sorted using samtools3. The sorted and aligned reads were counted into genes using htsec-counts 4a. In total, 35 million reads were aligned, and the alignment rate was more than 80 percent (Fig. S3). The IGV software (integrated genome viewer; Broad Institute) was used to view the aligned Illumina reads to the human genome and to visualize the data. Sequencing data have been deposited under https://www.ncbi.nlm.nih.gov/bioproject/PRJNA1140519 (BioSample accessions: SAMN42831966, SAMN42831967, SAMN42831968).

## Supplementary information

Supplemental rere

## Data Availability

RNA-seq NGS-sequencing data have been deposited under https://www.ncbi.nlm.nih.gov/bioproject/PRJNA1140519 (BioSample accessions: SAMN42831966, SAMN42831967, SAMN42831968)
