# Supplementary material for: Influenza A virus NS1 suppresses nuclear speckles promoted gene expression by inhibition of transcription
Source: Npj Viruses. 2025 May 30;3:46. doi: 10.1038/s44298-025-00124-x (PMC12122860; doi:10.1038/s44298-025-00124-x)
Supplement: Supplementary file 1 — Supplemental rere [file 44298_2025_124_MOESM1_ESM.pdf]

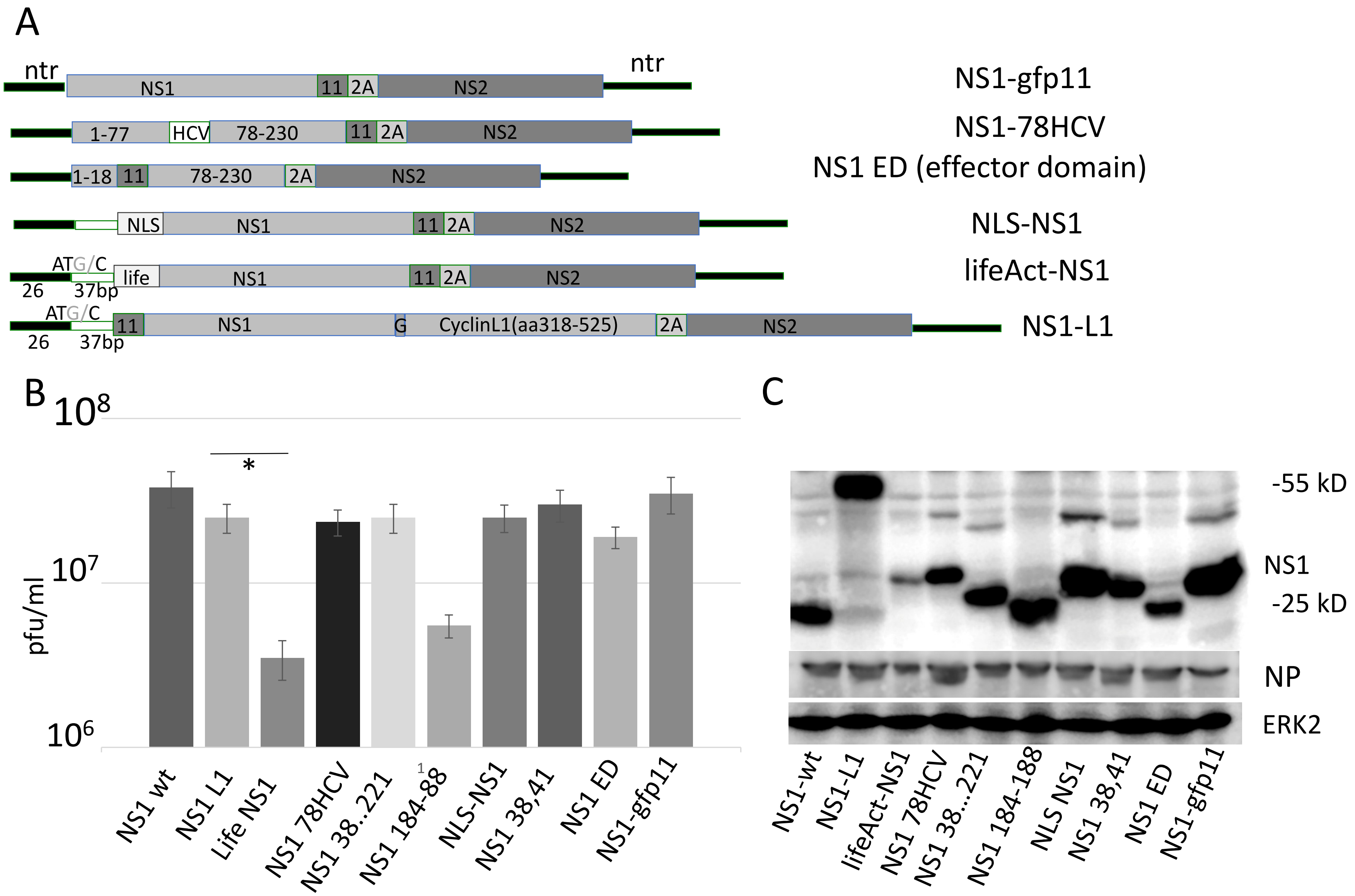
A
ntr
ntr
NS1
11
2A
NS2
NS1-gfp11
1-77
HCV
78-230
NS1-78HCV
1-18
NS1 ED (effector domain)
NLS
NLS-NS1
ATG/C
life
26
37bp
lifeAct-NS1
G
CyclinL1(aa318-525)
NS1-L1
B
10^8
10^7
10^6
pfu/ml
*
NS1 wt
NS1 L1
Life NS1
NS1 78HCV
NS1 38..221
NS1 184-88
NLS-NS1
NS1 38,41
NS1 ED
NS1-gfp11
C
-55 kD
NS1
-25 kD
NP
ERK2
NS1-wt
NS1-L1
lifeAct-NS1
NS1 78HCV
NS1 38...221
NS1 184-188
NLS NS1
NS1 38,41
NS1 ED
NS1-gfp11

Fig. S1

Figure S1: presentation of all recombinant IAVs used in this study. (A) Schematic representation of the constructs that encode the mutant IAV NS segments used in this study. NS1-ED (effector domain): NS1 aa1-18 separated by a gfp11 domain from aa 78-230; NS1 78HCV: NS1 separated by a peptide of the cleavage site of HCV NS3 used as spacer between aa position 77/78 of NS1; NS1-L1 (aa318-525): nuclear speckle localising domain of Cyclin L1; ntr: non-translated region; ATG/C: the first ATG was mutated to ATC to elongate the ntr, which was necessary to enhance virus titre; 37bp: length in base pairs of the sequence homologous to wild type NS segment used to elongate the ntr; 11: the 11th domain of GFP superfolder protein; life: yeast lifeAct peptide that mediates binding to actin filaments; 2A: 2A peptide from porcine teschovirus-1G: (Gly)3Ser peptide linker. (B) Titration of virus supernatants by plaque assay. pfu: plaque forming unit. NS1 38,41: IAV encoding NS1 (R38A, K41A); lifeNS1: IAV encoding lifeAct NS1 (R38A, K41A); NS184-188: IAV encoding NS1(GLEWN184-188 RFKRY); NS1 78HCV: IAV encoding HCV: peptide of the cleavage site of HCV NS3 between aa position 78/79 of NS1; NS1 38,41-L1: IAV encoding NS1 38,41 (R38A, K41A) fused to CyclinL1 (aa318-525); the experiments were repeated four times. *: $p<0,05$ (difference between Life NS1 38,41 and NS1 38,41-L1). (C) western blot: extracts of infected cells were run on a standard SDS-PAGE, blotted and the membrane probed with anti IAV-NS1 antibodies. The NS1 expression of all recombinant viruses used in this study is shown. For description of NS1 38,41, lifeAct NS1, NS184-188, NS1 78HCV, NS1-L1 see above. For NLS-NS1, NS1 38…221 see Fig. 1; NS wt: wild type NS1 without gfp11, NS1-gfp11: wild type NS1 with gfp11; all mutant NS1 variants are fused with GFP11 domain.

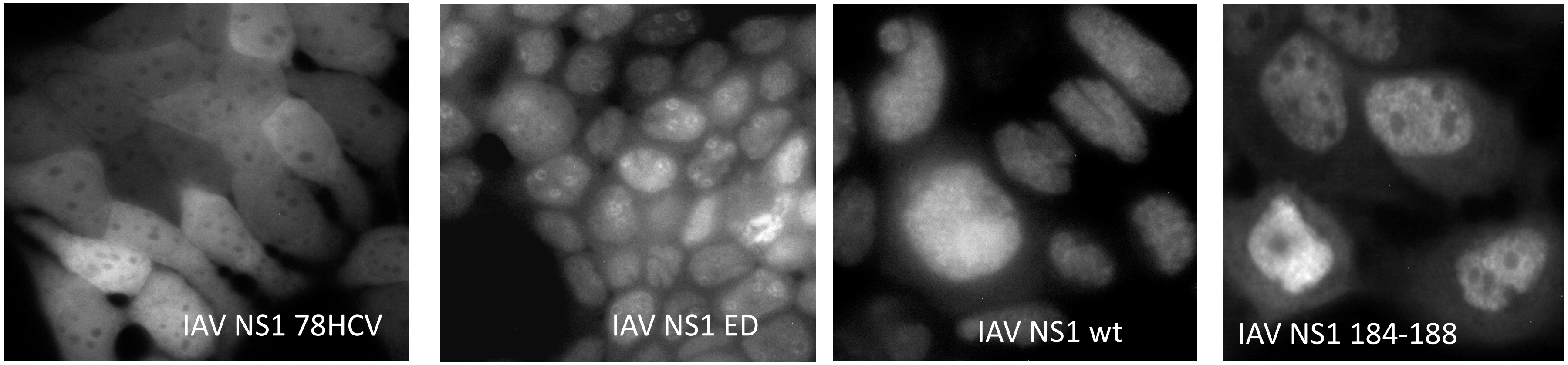

Figure S 2: Analysis of the subcellular localisation of recombinant NS1 upon infection by fluorescence microscopy. Cells expressing GFP 1-10 were infected with IAV encoding recombinant NS1 as described in Fig. 6. In the presence of NS1 self-assembly between GFP1-10 and the 11th domain of GFP fused to the corresponding NS1 protein (split GFP assay). Recombinant IAV are described in Fig. S1: 78-230, 1-18, 1-77: amino acid sequence of NS1 wild type; HCV: peptide of the cleavage site of HCV NS3 used as spacer peptide; CyclinL1 (aa318-525): nuclear speckle localising domain of Cyclin L1; HCV: peptide of the cleavage site of HCV NS3 used as spacer peptide; HCV: peptide of the cleavage site of HCV NS3 used as spacer peptide inserted between aa 78/79 of NS1; NS1-18-GFP11-78-230: aa 19-76 (RNA binding domain) are deleted and GFP11 domain inserted instead; NS1 184-188: mutant NS1 184-188 -GFP11(GLEWN184-188 RFKRY) unable to inhibit host transcription; wt: wild type

Fig. S2

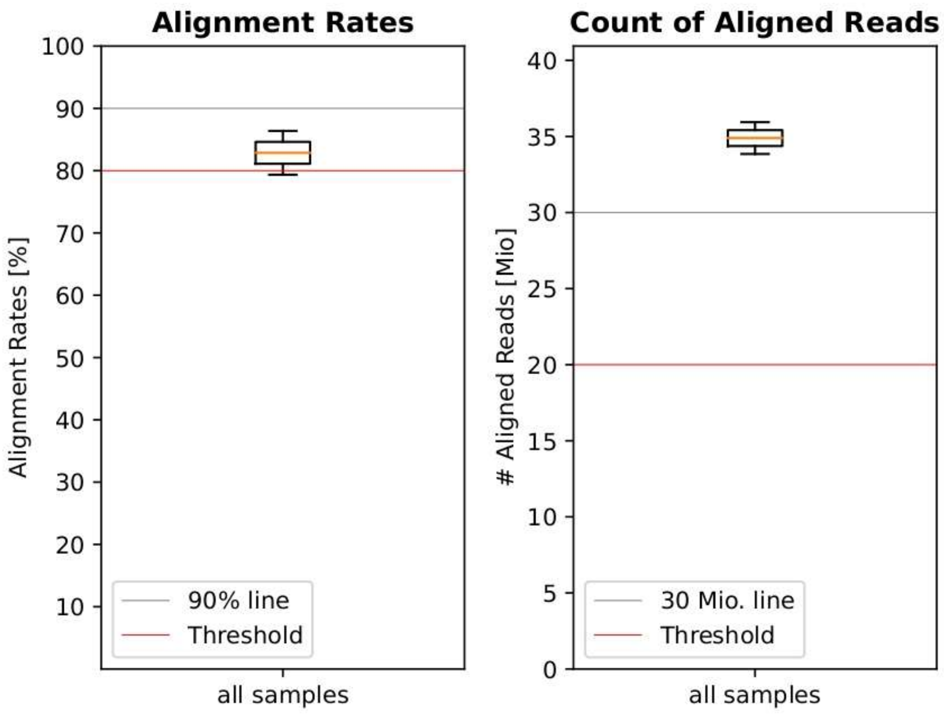

Figure S3: Boxplots are shown presenting basic summary statistics of the alignment (shown in fig. 9). Left panel: the percentage of reads mapped to the reference genome are indicated. For an ideal RNA-Seq library, this metric should be greater than or equal to 90 %. Alignment rates lower than 70 % may indicate serious issues with the data set. Right panel: count of aligned reads used for this study.

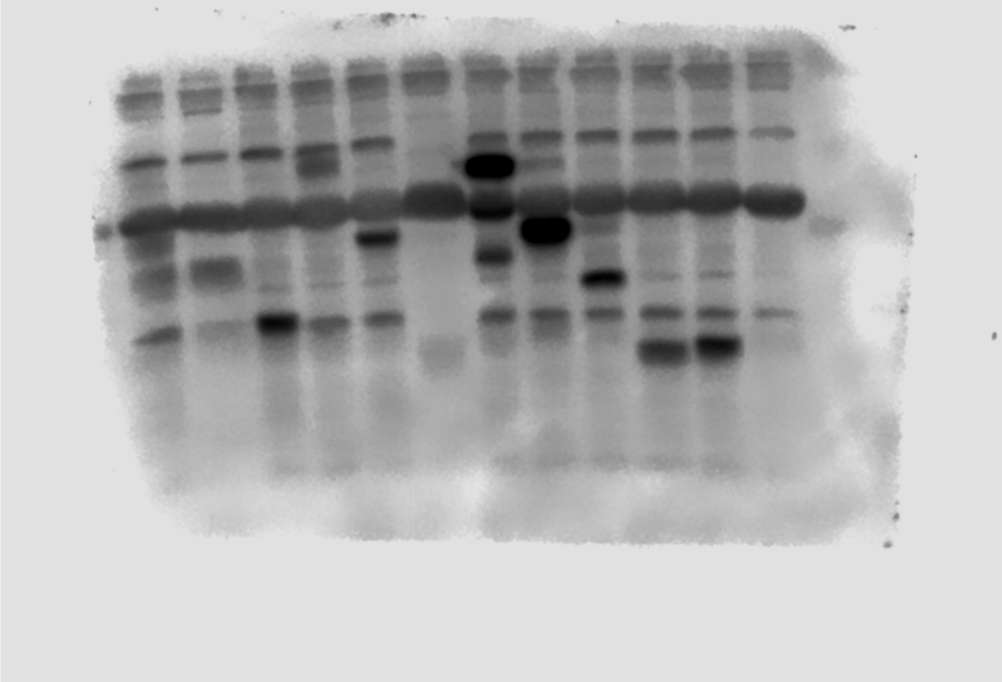

Fig. 3B original blot

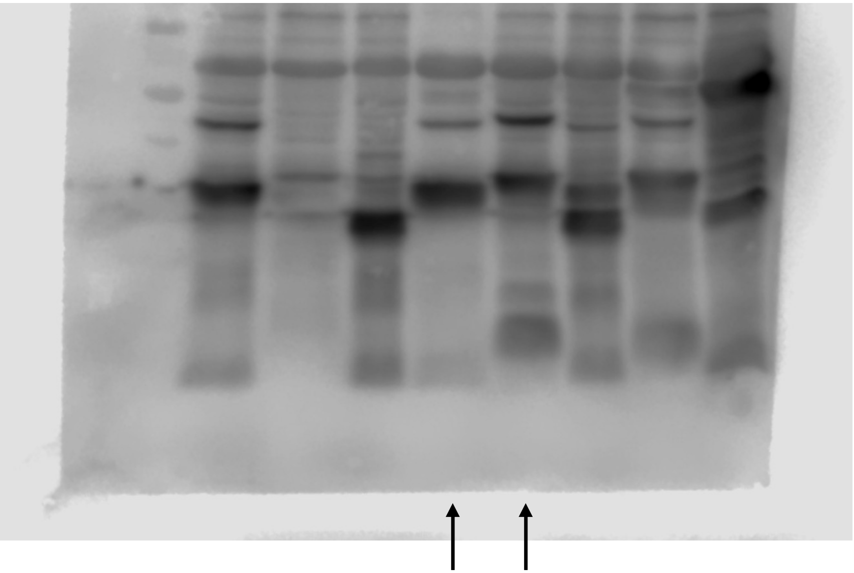

Fig. 10 C original blot

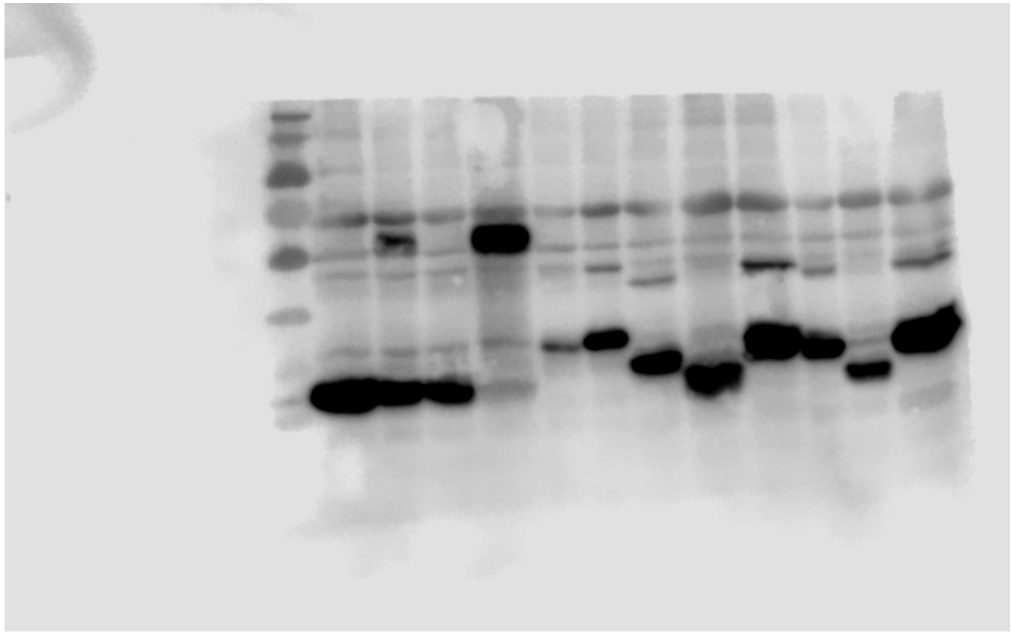

Fig. S1 C original blot
